# Compound Wumei Powder Inhibits the Invasion and Metastasis of Gastric Cancer via Cox-2/PGE2-PI3K/AKT/GSK3*β*/*β*-Catenin Signaling Pathway

**DOI:** 10.1155/2017/3039450

**Published:** 2017-11-21

**Authors:** Nai-Xia Ma, Wei Sun, Jian Wu, Shen-Lin Liu, Lei Weng, Yang-Qing Liu, Wen-Xiu Pu, Ting-Ting Wu, Xue-Lian Ding, Nan-Guang Huang, Pei-Qiu Zheng, Xi Zou

**Affiliations:** ^1^The Affiliated Hospital of Nanjing University of TCM, Jiangsu Province Hospital of TCM, Nanjing 210029, China; ^2^No. 1 Clinical Medical College, Nanjing University of Chinese Medicine, Nanjing, Jiangsu 210023, China; ^3^The Affiliated Hospital of Nanjing University of TCM, Liyang Hospital of TCM, Changzhou 213300, China

## Abstract

To explore the role of CWP in invasion and migration of gastric cancer cells and its underlying molecular mechanism, we performed the experiment in SGC-7901 cells both* in vitro* and* in vivo*. In the cell experiment, we evaluated cell proliferation by MTT assay. The results showed that CWP can inhibit the growth of SGC-7901 cells. The influence on cell migration and invasion was detected by wound-healing and Transwell invasion assays. The results showed that the abilities of invasion and migration are restrained in CWP group. Western blot showed that CWP can decrease the expression of Cox-2 and inhibit the PI3K/AKT/GSK3*β*/*β*-catenin signaling pathway. In the animal experiment, we observed that CWP had an inhibitory effect on the growth of xenograft tumors of nude mice. IHC assay, ELISA, RT-PCR assay, and Western blot assay were used to test relevant cytokines of Cox-2/PGE2-PI3K/AKT/GSK3*β*/*β*-catenin pathway. The results showed that CWP can suppress relevant cytokines of Cox-2/PGE2-PI3K/AKT/GSK3*β*/*β*-catenin pathway. In conclusion, we suggest that CWP inhibits the invasion and metastasis of SGC-7901 cells via Cox-2/PGE2-PI3K/AKT/GSK3*β*/*β*-catenin signaling pathway.

## 1. Introduction

Gastric cancer, which is one of the most commonly seen malignant tumors, is ranked second for cancer mortality in China [1]. It is generally acknowledged that surgery is the first priority for treatment of gastric cancer. However, the rate of tumor metastasis and recurrence of postsurgical patients remains high, as a result of invasion and migration of gastric cancer cells. Tumor recurrence and distant metastasis are common, which are the primary cause of the death of patients with gastric cancer [2]. Therefore, it is crucial to develop drugs that can prevent invasion and migration.

Compound Wumei Powder (CWP) is comprised of Wumei (*Prunus mume*, dried fruit of* Prunus mume *(Siebold and Zucc.)) and Wuweizi (*Fructus Schisandrae*, dried fruit of* Schisandra chinensis *(Turcz.) Baill.) equally. Both of them are classified to sour and astringent category in Chinese medicine. According to theory of Traditional Chinese Medicine (TCM) that “Herbs that are sour in flavor have the function of astringing and controlling capability,” CWP is used to secure qi and constrain gastric cancer. A large number of clinical researches from China have indicated that CWP can significantly improve patients' quality of life as well as prolonging survival time. We have successfully applied for a patent of the formula. Our previous studies have shown that CWP can inhibit invasion and metastasis of tumor cells. Nevertheless, the role of CWP in invasion and migration of gastric cancer cells and its underlying molecular mechanism have not yet been fully explored.

Overexpression of Cox-2 is closely related to the occurrence and development of gastric cancer [3, 4], for it contributes to proliferation, invasion, and migration of tumor cells, as well as enhancing tumor angiogenesis. Prostaglandin E_2_ (PGE2), which plays a crucial role in the occurrence and development of gastric cancer, is a kind of important cell growth factor and metabolite of Cox-2 catalyzing arachidonic acid [5]. PI3K/AKT/GSK3*β*/*β*-catenin signaling pathway exists in a variety of tumor cells [6–10]. Continuous activation of the signaling pathway can upregulate the expression of downstream genes and promote the invasion and metastasis of tumor cells. A series of studies have found that both Cox-2/PGE2 and PI3K/AKT/GSK3*β*/*β*-catenin signaling pathway can lead to tumor progression [11]. There may be a correlation in them and this needs to be elaborated.

In this study, we have performed experiments of CWP inhibiting invasion and metastasis of gastric cancer both* in vivo* and* in vitro* and revealed the role of Cox-2/PGE2-PI3K/AKT/GSK3*β*/*β*-catenin signaling pathway in the process.

## 2. Materials and Methods

### 2.1. Cells Culture and Reagent

Human gastric cancer cell line SGC-7901 was provided by Centre Laboratory of Jiangsu Provincial Hospital of TCM. It was cultured in RPMI-1640 medium supplemented with 10% fetal calf serum. The cells were incubated at 37°C, 5% CO2, and saturated humidity atmosphere and the medium was replaced every three days. The CWP consists of Wumei (*Prunus mume*, dried fruit of* Prunus mume *(Siebold and Zucc.)) and Wuweizi (*Fructus Schisandrae*, dried fruit of* Schisandra chinensis *(Turcz.) Baill.) Wumei and Wuweizi were sourced from Jiangsu Provincial Hospital of TCM. 100 g Wumei and 100 g Wuweizi were soaked for 30 minutes with 1000 mL of double-distilled water and then boiled with medium fire for 30 minutes, refluxed, and extracted. Repeat the boiling process with another 1000 mL of double-distilled water for 30 minutes once again. Two extracted solutions were then mixed and further vaporized to 50 ml by boiling. 4 g/ml represents the concentration of the raw herbs. The extract was stored in −20°C after sterilization and filtering through a 0.2 *μ*m filter. Celecoxib (Pfizer, J20150072) was diluted into a concentration of 40 mmol/L with anhydrous ethanol for use. PGE2 (number P5640) was purchased from Sigma-Aldrich (St. Louis, MO, USA). Insulin-like growth factor-1 (IGF-1, number ONS0414101) was purchased from R&D company. PI3K inhibitor LY294002 (number 9901S), primary antibodies against Cox-2, PI3K, P-AKT, AKT, P-GSK3*β*, GSK3*β*, *β*-catenin, MMP-2, MMP-7, and MMP-14, and secondary antibody were all purchased from Cell Signaling Technology (Beverly, MA, USA).

### 2.2. Compound Identification of Compound Wumei Powder

Components in CWP were characterized in liquid chromatograph-mass spectrometer/mass spectrometer (LC-MS/MS) instrumentation and conditions. Analysis was performed with Agilent HPLC 1200 system (Agilent, USA) consisting of a quaternary pump, an autosampler, and an online degasser. The chromatographic separation was performed on a Phenomenex Gemini C18, 3 *μ*m particle size, 110 Å, 100 mm (length) × 2.0 mm (I.D.) reversed phase analytical column. The mobile phase consisted of methanol-2 mM ammonium acetate (80 : 20; v/v) at a flow rate of 0.2 mL/min. The autosampler temperature was maintained at 4°C and the injection volume was 5 *μ*L. The total LC run time was 4 min with the column temperature kept at 35°C. Detection of analytes was performed on API 4000 tandem quadrupole mass spectrometer (Applied Biosystems, USA) with an electrospray ionization (ESI) interface in negative and positive ion mode. Multiple reaction monitoring (MRM) was used to monitor precursor to product ion transition of *m*/*z* 417.0→316.2 for schizandrin A, *m*/*z* 433.2→384.2 for schisandrin, *m*/*z* 193.0→133.9 for ferulic acid, and *m*/*z* 353.0→191.0 for chlorogenic acid. The analytical data were processed using Analyst software (version 1.4.1, Applied Biosystems). For analytes and IS, the source parameters were ion spray voltage of 4000 V, turbo heater temperature of 400°C, collision activation dissociation of 6 psi, and curtain gas of 20 psi. The compound-dependent parameters like declustering potential and collision energy were optimized at 110 V and 35 V for schizandrin A, 85 V and 25 V for schisandrin, 60 V and 25 V for ferulic acid, and 80 V and 30 V for chlorogenic acid, respectively. Quadrupole 1 and quadrupole 3 were maintained at unit resolution. Dwell time set was 150 ms for both analytes.

### 2.3. MTT Assay

Gastric cancer cells in logarithm phase were seeded in 96-well plates at the density of 6 × 103/well. After the cells were adherent to the walls, different concentration of the CWP in 0 mg/ml, 0.5 mg/ml, 1 mg/ml, 2 mg/ml, 4 mg/ml, and 8 mg/ml was handled for 12 h, 24 h, and 48 h, respectively. 120 *μ*l MTT (5 mg/ml) was added after removing the medium and incubated for 4 h in the incubator. Supernatant was removed and then 150 *μ*l dimethyl sulfoxide (DMSO) was added and shocked for 10 minutes. Absorbance under 490 nm was detected to calculate the absorption value. Inhibition rate = (1 − absorbance of test sample/absorbance of control sample) × 100%. The test was repeated for three times.

### 2.4. Animal Studies

20 BALB/c nude mice (number 201605969) were purchased from Charles River, Beijing, China. The mice received humane care according to the Nanjing Medical University Animal Care Committee guidelines. The mice were half male and female, 4 weeks old, weighing 16–20 g. They were fed in specific pathogen-free (SPF) environment by Animal Center of Nanjing Medical University. SGC-7901 cells were collected and cultured in logarithmic growth phase and the density was adjusted to 5 × 107/ml. Each mouse was inoculated with 0.1 ml of cell suspension on the right armpit after the disinfection. After ten days, the diameter of induration reached 9-10 mm; it suggested a successful model. The 20 mice were divided into four groups randomly: control group (NS 0.2 ml/10 g), low-dose group, medium-dose group, and high-dose group: orally gavaged CWP at 1 g/ml, 2 g/ml, and 4 g/ml, respectively. They were medicated for 11 days continuously. During the experimental period, the daily diet and active status of the mice were normal. The weight of the mice and short-axis (*a*) and long-axis (*b*) diameters of the tumor were recorded every other day, the volume of the tumor was calculated (*V* = *a* × *b*^2^/2), and the growth curve of the tumor was drawn. The blood was taken from the orbit after the last administration, and the concentration of PGE2 in the blood was measured. The tumors peeled from the nude mice were weighed and the inhibition rate of each group was calculated. The inhibition rate (%) = (1 − average weight of test sample/average weight of control sample) × 100%.

### 2.5. Immunohistochemistry Assay

Paraformaldehyde-fixed tumor tissue was embedded with paraffin and cut into sections. The sections were mounted on slides and soaked in xylene for 5 min twice, soaked in anhydrous ethanol, 95% ethanol, 85% ethanol, and 70% ethanol for 5 min, and soaked in Phosphate-Buffered Saline (PBS) for 3 min three times, respectively. The sections were boiled in 10 mM sodium citrate buffer (pH 6.0) for 5 min and cooled for 30 min, followed by incubation in 3% hydrogen peroxide for 15 min and blocking with normal goat serum for 30 min. Sections were incubated with primary antibodies (Cox-2, MMP-2, MMP-7, and MMP-14) and then washed with PBST buffer and incubated with HRP conjugated anti-rabbit IgG (Boshide Biological Technology Co., Ltd., Wuhan, China). The staining of the sections was performed using hematoxylin. Three views were chosen randomly of each group to take pictures and make sure the light of the background is constant. Image-Pro was adopted to analyze each picture to get its integrated optical density (IOD) and area. Mean density (IOD/area) was used to analyze the expression of protein.

### 2.6. Enzyme-Linked Immunosorbent Assay (ELISA)

Put the blood of nude mice into Ethylenediaminetetraacetic acid (EDTA) anticoagulative tube. After standing for 1 h in room temperature, centrifugation (3000 rpm for 20 min), collecting the serum, and measuring PGE2 concentration using an ELISA kit (Affinity BioReagents Co., Ltd.), Take the serum into ELISA plate, and perform incubation at 37°C for 1 h and washing by buffer for 3 min × 3 times. Addi the enzyme-labeled antibody and perform incubation for 1 h and washing again. Add hydrogen peroxide urea solution at 37°C in the dark for 5 min and add stop buffer. Measure the optical density (OD) value under 450 nm within 30 min. Work out the concentration of the sample according to the standard concentration and the OD value.

### 2.7. Transwell Assay

Matrigel (50 ul, 1 : 4 dilution by serum-free medium) was vertically added to the inside bottom of the Transwell chambers. Fibronectin (FN) (30 ul, diluted to 70 ug/ml by sterile water) was dripped onto the outside bottom of the chambers evenly, and the chambers were dried in the clean bench for use. The cells were starved in serum-free RPMI-1640 for 24 h. Afterwards, they were digested and resuspended with serum-free medium to 5 × 10^6^/ml. 100 ul of cell suspension was added to the chamber and drugs were administered at the same time. The chambers were placed in 24-well plates with RPMI-1640 medium and cultured in the incubator. After 24 h, the chambers were taken off and nonpenetrative cells were washed on the top chamber by PBS. Migrated cells were fixed with 95% ethanol and stained by crystal violet. Photos of each group were taken with the microscope and counted randomly.

### 2.8. Wound-Healing Assay

The SGC-7901 cells were seeded in 6-well plates. When they were adherent to the walls up to 70%, a straight line was drawn on the bottom of the plates with a pipette tip and then washed with PBS three times lightly. The pictures were taken with the microscope and the instance between wound boundaries was recorded. The medium was changed to the serum-free RPMI-1640 medium; different drugs were administered in each group and pictures were taken again after incubating for 24 h.

### 2.9. Western Blot Assay

The protein was extracted from the tumor of mice and the SGC-7901 cells. The concentration of protein lysates from cultured cells and tumor was tested by Bradford Protein Assay Kit. The protein lysates were separated on sodium dodecyl sulfate polyacrylamide gel electrophoresis (SDS-PAGE) and then transferred to polyvinylidene fluoride (PVDF) membranes. Membranes were blocked with 5% BSA for an hour and incubated with primary antibodies at −4°C overnight. The membranes were taken off and washed by Tris-buffered saline with Tween-20 (TBST) for 3 times × 5 min, followed by incubation with secondary antibody for 1 h at room temperature. The membranes were washed by TBST for 4 times × 5 min. Signals were observed under the Image Studio system, version 3.1.4.

### 2.10. RNA Extraction and Real-Time Quantitative Analysis

Total RNA was extracted from the cells by TRIzol (Takara, Japan). The reverse transcription was performed with the RT-PCR kit. Amplified reaction was performed in the 7500 Fast System. The primer was synthesized by Invitrogen Co. Sequences of the primer were shown as follows: A 273 bp fragment of Cox-2 was generated using sense (5′-GATGATTGCCCGACTCCCTT-3) and antisense (5′-GAAAAGGCGCAGTTTACGCT-3′) primer. A 207 bp fragment of *β*-actin was generated using sense (5′-TGTCTCTGGACGGCAGCTAT-3′) and antisense (5′-TTGCATCTCCTTGAGTTTGGC-3′) primer.

### 2.11. Statistical Analysis

All the statistical analyses were performed with SPSS 18.0 software. All data were expressed as means ± standard deviation (SD). Statistically significant differences between groups were analyzed by *t*-test and one-way ANOVA. Statistical significance was set at *P* < 0.05.

## 3. Results

### 3.1. Compound Identification of CWP by LC-MS/MS Technology

The chemical composition of the CWP was determined through LC-MS/MS. The major components in CWP were schisandrin and chlorogenic acid, with high concentrations of 140.65 *μ*g/ml and 123.12 *μ*g/ml, respectively. Schizandrin A and ferulic acid were also detected in CWP with concentrations of 8.32 *μ*g/ml and 1.89 *μ*g/ml, respectively (Table 1).

### 3.2. CWP Inhibited the Proliferation of Gastric Cancer Both* In Vitro* and* In Vivo*

**Figure 1:**
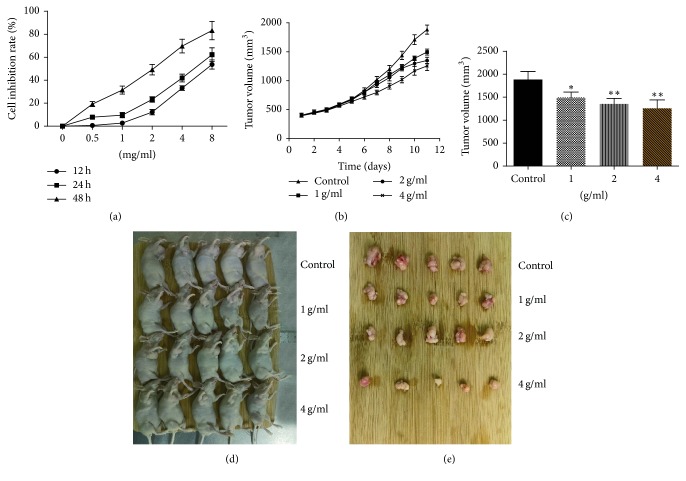
CWP can inhibit the proliferation of gastric cancer cell SGC-7901. (a) SGC-7901 cells in logarithmic growth phase were treated with different dosages of CWP for 12 h, 24 h, and 48 h, respectively. The inhibition of each group of cells was detected with MTT assay. (b) CWP inhibited the growth of implanted tumor; tumor volumes of each group were recorded every other day. (c) After 11 days of administration, the transplanted tumors were stripped and the volume of each tumor was measured. (d) Nude mice were subcutaneously implanted with 5 × 10^6^ SGC-7901 cells. The mice were divided into control group, CWP 1 g/ml, CWP 2 g/ml, and CWP 4 g/ml after tumor had been completely developed. (e) The tumors of each group were peeled out after 11 days. Data are expressed as the mean ± SD of 5 mice (^*∗*^*P* < 0.05 compared with control group; ^*∗∗*^*P* < 0.01 compared with control group).

We first demonstrated that CWP had an inhibitory effect on the growth of human gastric cancer cell SGC-7901* in vitro*. The gastric cancer cells were treated with CWP 0.5 mg/ml, 1 mg/ml, 2 mg/ml, 4 mg/ml, and 8 mg/ml, respectively for 12 h, 24 h, and 48 h. The inhibitory effects of drugs on the cells were time-dependent (*P* < 0.01); the IC_50_ values of 12 h, 24 h, and 48 h were 7.28 mg/ml, 5.60 mg/ml, and 2.00 mg/ml, respectively (Figure 1(a)). Animal experiments showed that CWP had an inhibitory effect on the growth of xenograft tumors of SGC-7901 cells in nude mice. The mice were randomly divided into control group, low-dose group, medium-dose group, and high-dose group. The tumor inhibition rates of each group were 31.83%, 34.83%, and 55.26% compared with the control group and the status of nude mice in each group was good. At the end of the experiment, we found that CWP inhibits the tumor volume in a drug dosage-dependent manner (*P* < 0.01) (Figures 1(b)–1(e)).

### 3.3. CWP Inhibited the Invasion and Migration of SGC-7901 Cells* In Vitro*

**Figure 2:**
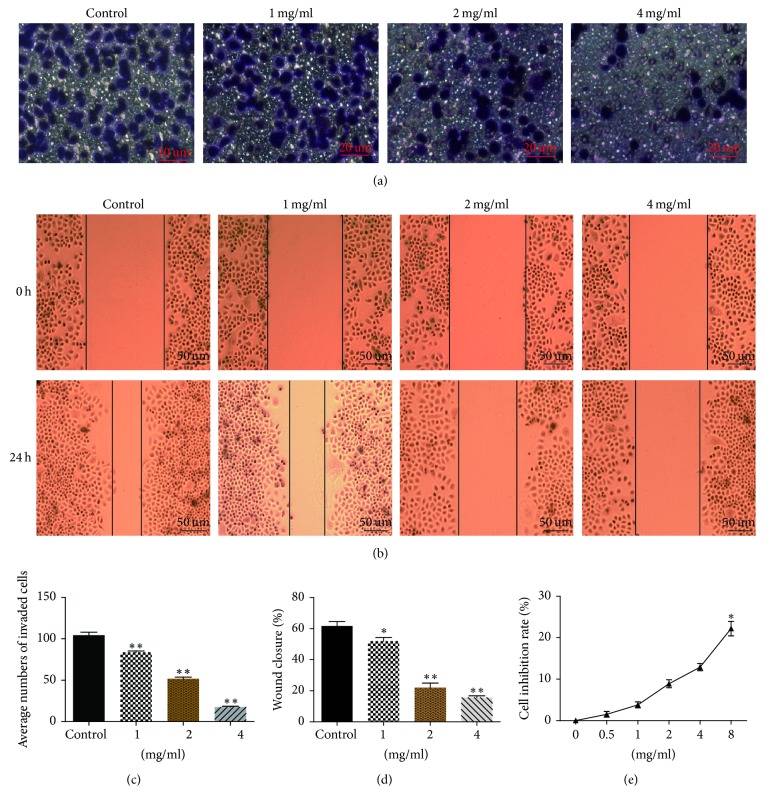
CWP can inhibit the invasion and migration of SGC-7901 cells. (a) Transwell experiments were performed on SGC-7901 cells for 24 h with different concentrations of CWP. The cells were inserted into the lower chamber through the basement membrane, and the basement membrane was stained and observed under a microscope. (b) SGC-7901 cells seeded on six-well plates were divided into four groups. After administration for 24 hours, the cells migration was recorded. (c) The histogram shows the number of SGC-7901 cells passing through the 8 *μ*m pore size into the lower chamber. (d) The histogram shows the percentage of wound healing in SGC-7901 cells after 24 h. (e) SGC-7901 cells were treated with different dosages of CWP for 24 h on the condition of wound-healing assay. The inhibition of each group was detected with MTT assay. Data are expressed as the mean ± SD of three experiments (^*∗*^*P* < 0.05 compared with control group; ^*∗∗*^*P* < 0.01 compared with control group).

We next performed Transwell and wound-healing assays to explore the impact of CWP on the invasion and migration of SGC-7901 cells. As shown, CWP could significantly inhibit the invasion and migration of SGC-7901 cells in a dosage-dependent manner (*P* < 0.01) (Figures 2(a)–2(d)).

To eliminate the possibility that CWP treatment may inhibit growth and thereby influence the rate of migration, SGC-7901 cells were planked and treated as wound-healing assay. 100 ul suspension with 6 × 103 SGC-7901 cells was seeded on 96-well plates in RPMI-1640 medium containing 10% fetal calf serum, and cells were cultured for 8 h to form a confluent monolayer. Then medium was removed and cells were treated with different doses of CWP in serum-free medium for 24 h. As shown in Figure 2(e), the data indicated that less than 4 mg/ml CWP exerted few influences on proliferation of SGC-7901 cells (*P* > 0.05), and the max growth inhibitory rate for cells incubated with 8 mg/ml CWP merely reached 12% (*P* < 0.05). Taking these above findings together, it could be illustrated that CWP exhibited minor inhibitive effects on the viability of SGC-7901 cells but could observably suppress the capability of migration.

### 3.4. CWP Inhibited the Expression of Cox-2* In Vitro* and* In Vivo* and Reduced the Concentration of PGE2 in Serum

**Figure 3:**
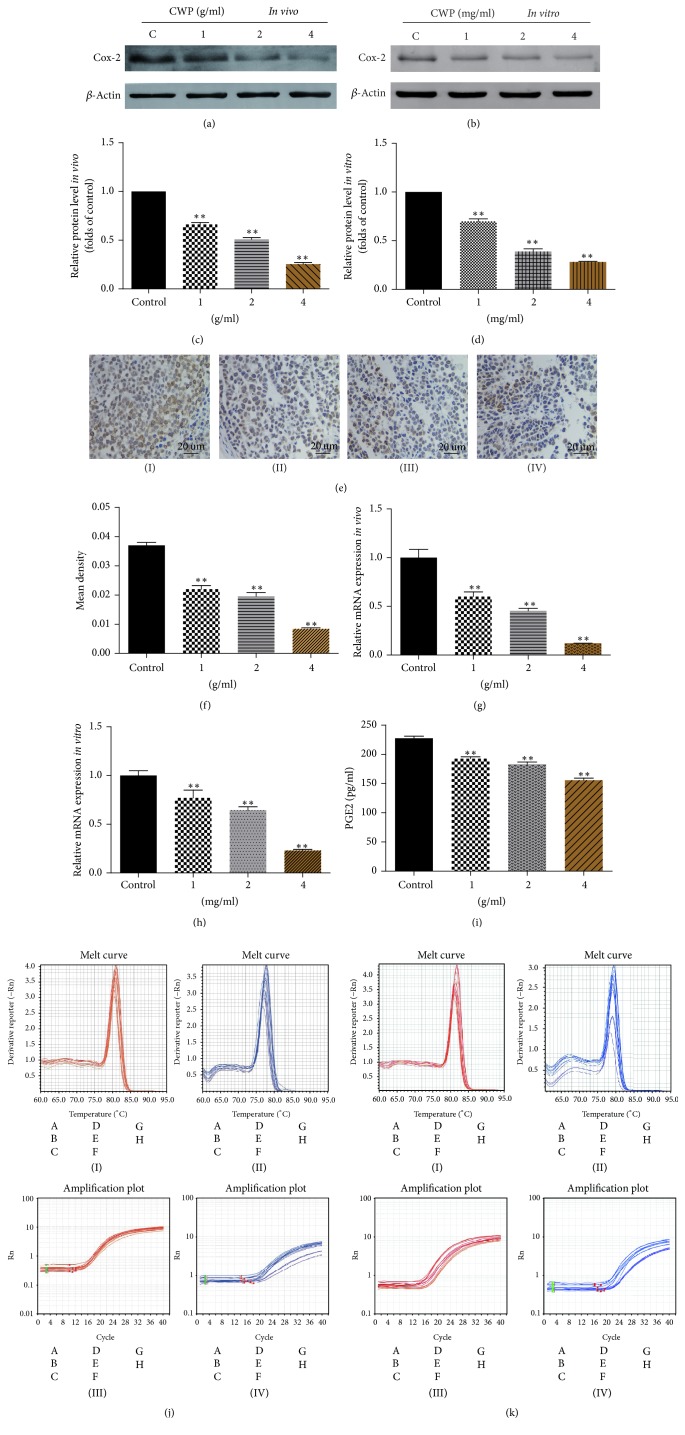
CWP inhibited the expression of Cox-2, MMP-7, and PGE2. (a) Extracts from transplanted tumor were analyzed for the expression of Cox-2. (b) The histogram shows the expression of Cox-2 compared to the control group* in vivo*. (c) Extracts from SGC-7901 cells were analyzed for the expression of Cox-2. (d) The histogram shows the expression of Cox-2 compared to the control group* in vitro*. (e) Immunohistochemistry assay was used to detect the expression of Cox-2 of transplanted tumor; expression levels are higher in control group. (f) The histogram shows the mean density (average density) of Cox-2. (g) The histogram shows the mRNA expression of Cox-2 of the tumor by RT-PCR* in vivo*. (h) The histogram shows the mRNA expression of Cox-2 of the tumor by RT-PCR* in vitro*. (i) The histogram shows the level of PGE2 in nude mice by ELISA method. (j) The images show melt curve and amplification plot of (g). (k) The images show melt curve and amplification plot of (h) ((I) and (II) show the melt curve of *β*-actin and Cox-2; (III) and (IV) show the amplification plot of *β*-actin and Cox-2). Data are expressed as the mean ± SD of three experiments (^*∗∗*^*P* < 0.01 compared with control group).

Then, we studied the potential mechanism that CWP can inhibit the invasion and migration of SGC-7901 cells. We found that the gene expression levels of Cox-2 decreased both* in vivo* and* in vitro*, while the concentrations of drugs were increased (Figures 3(a)–3(h)). ELISA method was used to demonstrate that the content of PGE2 in serum of nude mice was decreased in a dose-dependent manner (Figure 3(i)).

### 3.5. CWP Inhibited PI3K/AKT/GSK3*β*/*β*-Catenin Signaling Pathway Both* In Vivo* and* In Vitro*

**Figure 4:**
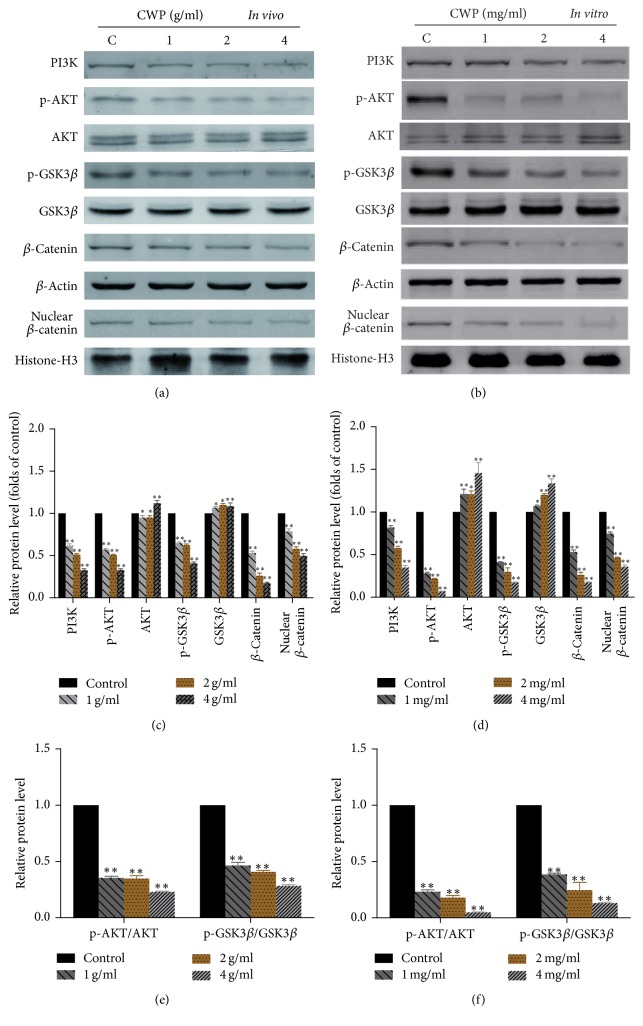
CWP inhibits PI3K/AKT/GSK3*β*/*β*-catenin signaling pathway. (a) Extracts from transplanted tumor of different groups were analyzed for the expression of PI3K, p-AKT, AKT, p-GSK3*β*, GSK3*β*, *β*-catenin, and nuclear *β*-catenin. (b) Extracts from SGC-7901 cells of different groups were analyzed for the expression of PI3K, p-AKT, AKT, p-GSK3*β*, GSK3*β*, *β*-catenin, and nuclear *β*-catenin. (c) The histogram shows the relative expression of each protein in (a). (d) The histogram shows the relative expression of each protein in (b). (e) Histogram indicates the ratio of p-AKT/AKT and p-GSK3*β*/GSK3*β* of (a). (f) The histogram indicates the ratio of p-AKT/AKT and p-GSK3*β*/GSK3*β* of (b). Data are expressed as the mean ± SD of three experiments (^*∗*^*P* < 0.05 compared with control group; ^*∗∗*^*P* < 0.01 compared with control group).

**Figure 5:**
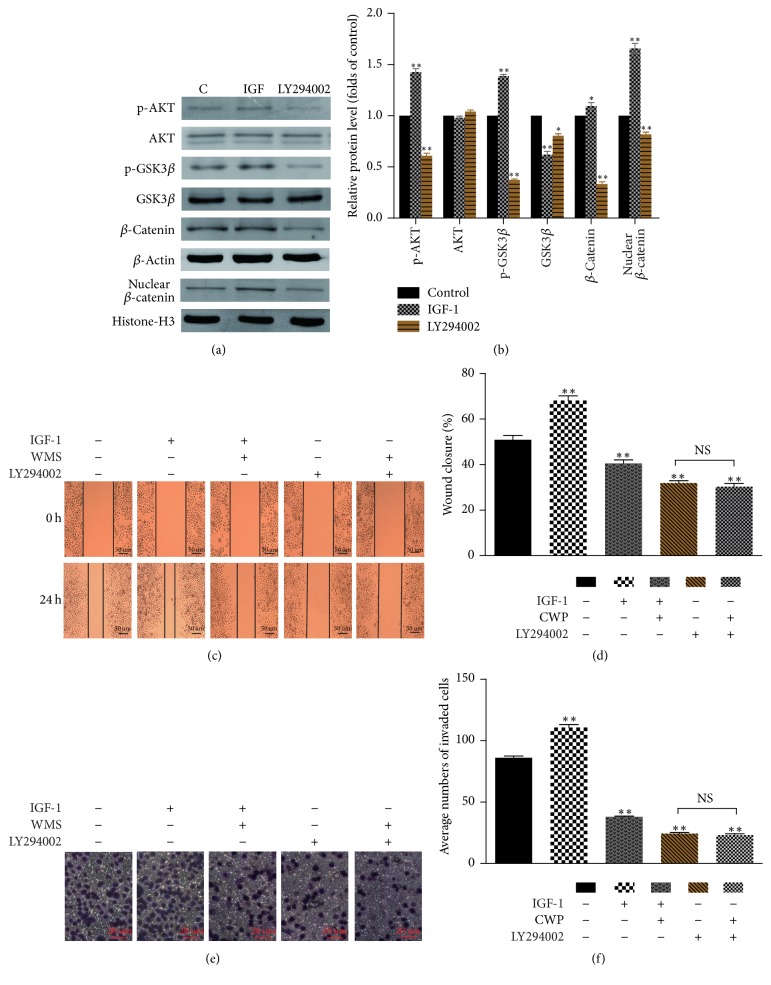
CWP inhibits PI3K/AKT/GSK3*β*/*β*-catenin signaling pathway. (a) The expressions of p-AKT, AKT, p-GSK3*β*, GSK3*β*, and *β*-catenin were detected after treatment with IGF for 2 h and LY294002 for 24 hours. (b) The histogram shows the relative expression of protein in (a). (c) Observation of cell migration after the cells were treated with PI3K/AKT agonist IGF-1 and inhibitor LY294002. (d) The histogram shows the percentage of wound healing in SGC-7901 cells after administration. (e) Observation of cell invasion after the SGC-7901 cells were treated with IGF-1 and LY294002. (f) The histogram shows the number of SGC-7901 cells passing through the 8 *μ*m pore size into the lower chamber. Data are expressed as the mean ± SD of three experiments (^*∗*^*P* < 0.05 compared with control group; ^*∗∗*^*P* < 0.01 compared with control group; NS means no significance).

In order to elucidate the mechanism of the action of CWP on cell migration and invasion, we found that the cell signaling pathway was affected by CWP. Western blot showed that, compared with the control group, the phosphorylation levels of AKT (p-AKT) and GSK3*β* (p-GSK3*β*) and the expression of *β*-catenin in the cytoplasm and nucleus were decreased in experimental group (*P* < 0.01) (Figures 4(a)–4(f)). The expressions of p-AKT, p-GSK3*β*, and *β*-catenin were declined after administration of LY294002, which is a specific inhibitor of PI3K/AKT (*P* < 0.01). On the contrary, the expression increased after administration of IGF-1 (*P* < 0.01). Accordingly, ability of invasion and migration was suppressed by LY294002, while it was strengthened by IGF-1 (Figures 5(a)–5(f)).

### 3.6. Cox-2/PGE2 Promoted the Invasion and Migration of SGC-7901 Cells and Regulates PI3K/AKT/GSK3*β*/*β*-Catenin Signaling Pathway

**Figure 6:**
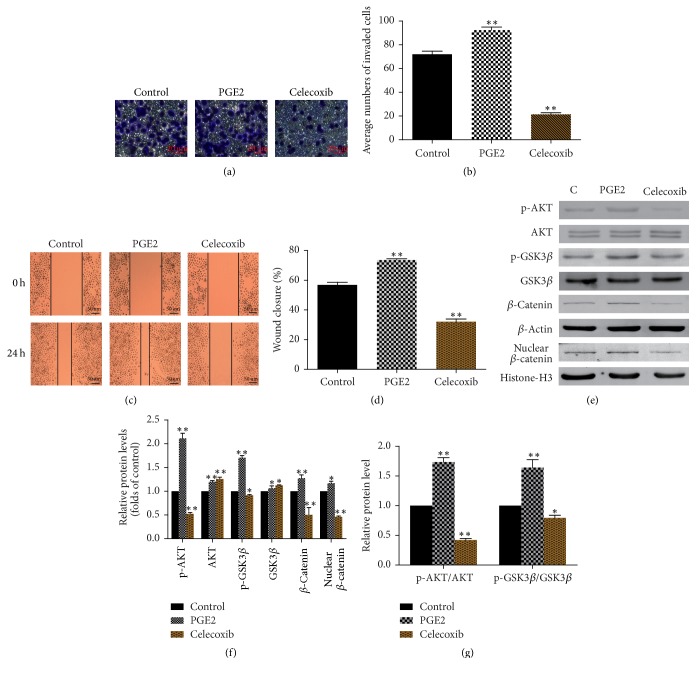
Cox-2/PGE2 promotes the invasion and migration and regulates PI3K/AKT/GSK3*β*/*β*-catenin signaling pathway. (a) Observation of cell invasion after the SGC-7901 cells were treated with PGE2 and Celecoxib. (b) The histogram shows the number of SGC-7901 cells passing through the 8 *μ*m pore size into the lower chamber. (c) Observation of cell migration after the cells were treated with PGE2 and Celecoxib. (d) The histogram shows the percentage of wound healing in SGC-7901 cells after administration. (e) Extracts from SGC-7901 cells treated with PGE2 and Celecoxib were analyzed for the expression of p-AKT, AKT, p-GSK3*β*, GSK3*β*, *β*-catenin, and nuclear *β*-catenin. (f) The histogram shows the relative expression of each protein in (e). (g) The histogram shows the relative ratios of p-AKT/AKT and p-GSK3*β*/GSK3*β* in each group. Data are expressed as the mean ± SD of three experiments (^*∗*^*P* < 0.05 compared with control group; ^*∗∗*^*P* < 0.01 compared with control group).

We speculated that the inhibition of invasion and migration by CWP may be related to the downexpression of Cox-2/PGE2. In order to verify our hypothesis, we used Celecoxib, selective inhibitor of Cox-2, to inhibit the expression of Cox-2. We found that cell invasion and migration were significantly suppressed compared with control group (*P* < 0.01). Further, we also treated cells with PGE2. The results showed that the invasion and migration abilities of SGC-7901 cells were significantly enhanced after being treated with PGE2 (*P* < 0.01). The expression levels of p-AKT/AKT, p-GSK3*β*/GSK3*β*, and *β*-catenin were downregulated in Celecoxib group and upregulated in PGE2 group. The PI3K/AKT/GSK3*β*/*β*-catenin signaling pathway was activated by PGE2 (Figures 6(a)–6(g)).

### 3.7. CWP Inhibited the Invasion and Metastasis of SGC-7901 Cells via Cox-2/PGE2-PI3K/AKT/GSK3*β*/*β*-Catenin Signaling Pathway

**Figure 7:**
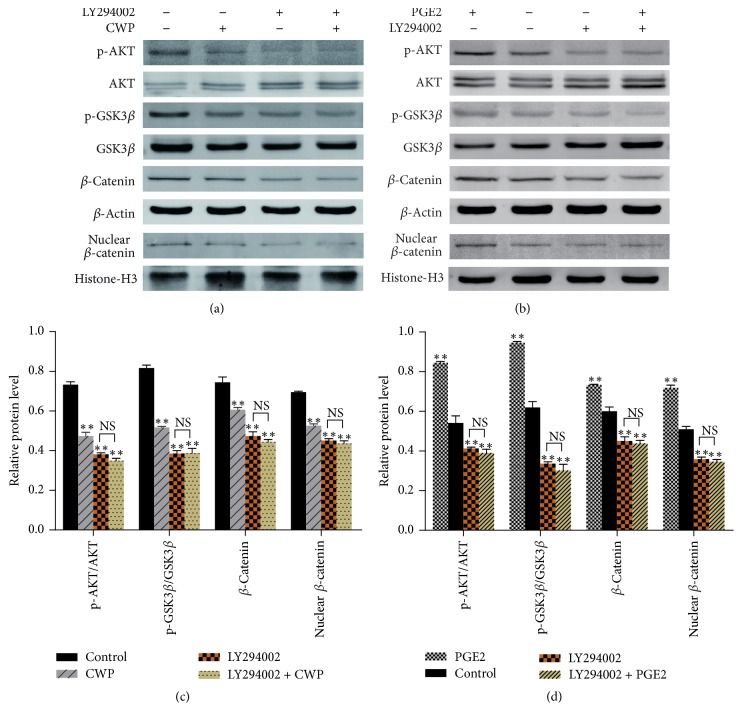
CWP can inhibit the invasion and metastasis of SGC-7901 via Cox-2/PGE2-PI3K/AKT/GSK3*β*/*β*-catenin signal axis. (a) Extracts from SGC-7901 cells of different groups treated with LY294002 and CWP were analyzed for the expressions of p-AKT/AKT, p-GSK3*β*/GSK3*β*, *β*-catenin, and nuclear *β*-catenin. (b) Extracts from SGC-7901 cells of different groups treated with PGE2 and LY294002 were analyzed for the expressions of p-AKT/AKT, p-GSK3*β*/GSK3*β*, *β*-catenin, and nuclear *β*-catenin. (c) The histogram shows the relative expression of each protein in (a). (d) The histogram shows the relative expression of each protein in (b). Data are expressed as the mean ± SD of three experiments (^*∗∗*^*P* < 0.01 compared with control group; NS means no significance).

As shown in Figure 6(e), PGE2 could upregulate p-AKT/AKT, p-GSK3*β*/GSK3*β*, *β*-catenin, and nuclear *β*-catenin, while Celecoxib, selective inhibitor of Cox-2, could upregulate the expression level of p-AKT/AKT, p-GSK3*β*/GSK3*β*, *β*-catenin, and nuclear *β*-catenin.* Thus, we suggest that Cox-2/PGE2 is upstream of PI3K/AKT/GSK3β/β-catenin signaling*. Our study also indicated that CWP reduced the expression of Cox-2/PGE2 and inhibited PI3K/AKT/GSK3*β*/*β*-catenin signaling pathway. We hypothesized that the potential mechanism of CWP repressing invasion and migration of SGC-7901 was related to its inhibitory effect on Cox-2/PGE2-PI3K/AKT/GSK3*β*/*β*-catenin signaling axis. To verify our hypothesis, the inhibitor LY294002 was used to suppress the signaling pathway; we found that CWP could not decrease the expression levels of p-AKT/AKT, p-GSK3*β*/GSK3*β*, and *β*-catenin after treatment with LY294002. There was no statistical significance between group LY294002 and group LY294002 + CWP (*P* > 0.05). PGE2 could upregulate p-AKT/AKT, p-GSK3*β*/GSK3*β*, *β*-catenin, and nuclear *β*-catenin, while pretreatment with LY294002, the stimulatory effect of PGE2, could be blocked obviously. There was also no statistical significance between group LY294002 and group LY294002 + PGE2 (*P* > 0.05) (Figures 7(a)–7(d)). The results showed that CWP could inhibit the invasion and metastasis of gastric cancer cells through Cox-2/PGE2-PI3K/AKT/GSK3*β*/*β*-catenin signal pathway.

### 3.8. CWP Can Suppress the Expressions of MMP-2, MMP-7, and MMP-14

**Figure 8:**
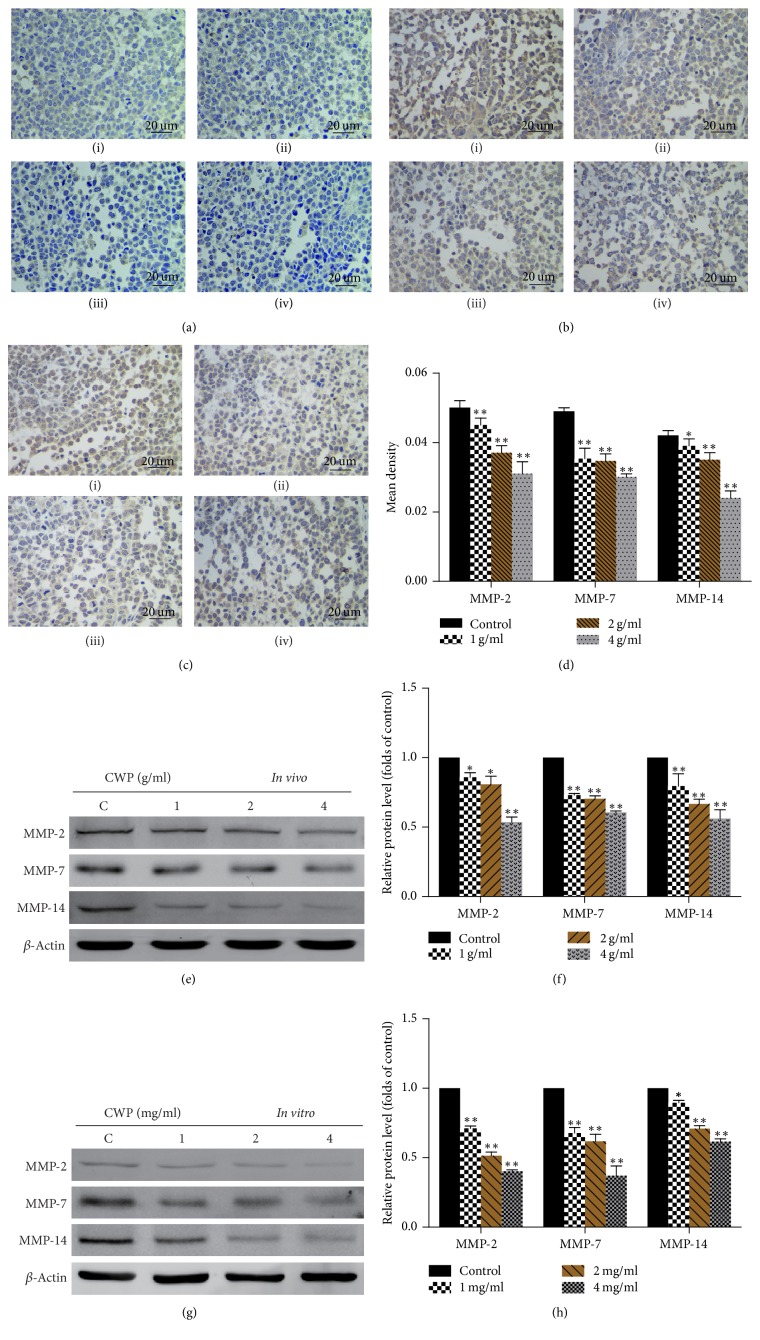
CWP can suppress the expressions of MMP-2, MMP-7, and MMP-14. (a)–(c) Immunohistochemistry assay was used to detect the expressions of MMP-2, MMP-7, and MMP-14 of (i) (control), (ii) (1 g/ml), (iii) (2 g/ml), and (iv) (4 g/ml) transplanted tumor; expression levels are higher in control group. (d) The histogram shows the mean density (average density) of MMP-2, MMP-7, and MMP-14. (e) Extracts from transplanted tumor were analyzed for the expressions of MMP-2, MMP-7, and MMP-14. (f) The histogram shows the expressions of MMP-2, MMP-7, and MMP-14 compared to the control group* in vivo*. (g) Extracts from SGC-7901 cells were analyzed for the expressions of MMP-2, MMP-7, and MMP-14; (h) The histogram shows the expressions of MMP-2, MMP-7, and MMP-14 compared to the control group* in vitro* (^*∗*^*P* < 0.05 compared with control group; ^*∗∗*^*P* < 0.01 compared with control group).

MMPs consist of a family of more than 25 neutral Zn^2+^-binding proteinases. MMP-2, MMP-7, and MMP-14 are members of matrix metalloproteinase family, which play an important role in gastric cancer invasion and migration. Overexpression of MMP-2 is related to distant metastasis of gastric cancer patients [12]. Restoration of MMP-7 can increase invasive activity of gastric cancer [13]. Overexpressed MMP-14 in gastric cancer may enhance local cell invasion and metastasis. Recent study showed that MMP-2 and MMP-14 could be candidates for molecular markers of gastric cancer [14–16]. Thus, we detected the expressions of MMP-2, MMP-7, and MMP-14. Immunohistochemistry and Western blot showed that, compared with the control group, levels of MMP-2, MMP-7, and MMP-14 declined in a dosage-dependent manner (Figures 8(a)–8(f)).

## 4. Discussion

Recurrence and metastasis of malignant tumor have become a major threat to human health and account for a high rate of mortality. Relapse rates of patients with gastric cancer who have radical resection are still high within five years. Therefore, effective strategies are urgently required to prevent metastasis of gastric cancer and increase survival rate. In addition to surgery and chemotherapy, TCM treatment has become a popular and promising alternative therapy. It has great advantage in the treatment of cancer in long-term clinical practice. Exploration of antitumor theory in terms of TCM is therefore necessary. Our experimental data have demonstrated that CWP can inhibit migration and invasion of SGC-7901 cells and we have raised possible mechanism for the sake of future study.

Cox-2 can catalyze arachidonic acid into PGE2. Many studies have shown that Cox-2 is overexpressed in a variety of malignant tumors [3, 4, 17, 18]. Binding of PGE2 to the E-series of Prostaglandin Receptors (EP receptors) on the cell membrane activates the PI3K/AKT pathway, followed by phosphorylation of GSK3*β* [19–21]. PGE2 plays a crucial role in promoting tumor angiogenesis, cell growth, migration, and invasion [20]. Evidences are mounting that long-term administration of Cox-2 inhibitors such as nonsteroidal anti-inflammatory drugs can reduce metastasis and recurrence of cancer notably [22, 23]. Our study indicated that CWP can inhibit the expression of Cox-2 and PGE2 both* in vivo* and* in vitro* in a dose-dependent manner. Cell experiments have shown that the abilities of invasion and migration are enhanced in the PGE2 group, while they are restrained in Celecoxib group. Compared with PGE2 group, CWP + PGE2 could weaken the ability of PGE2 in terms of invasion and migration of SGC-7901 cells. Therefore, it is necessary to explore whether the mechanism of CWP inhibiting tumor recurrence and metastasis is related to properties of PGE2.

**Figure 9:**
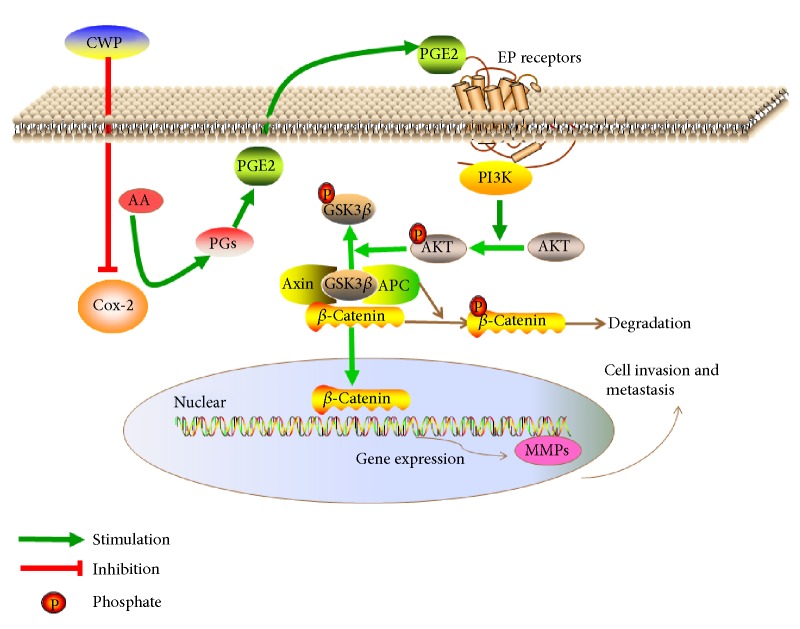
A hypothetical illustration for the role of CWP. PGE2, the main catalyzed product of Cox-2 from arachidonic acid, could bind to the EP receptor on the cell membrane, thereby activating the PI3K. Next, phosphorylation of AKT on Ser473 sites generated by PI3K and p-AKT can inhibit the activity of GSK3*β* by phosphorylating it. *β*-Catenin was accumulated in the cytoplasm and translocated into the nucleus, thereby activating downstream target genes MMPs.

PI3K/AKT/GSK3*β*/*β*-catenin signaling is activated in most cancers, especially in gastrointestinal tumors [21, 24–26]. PI3K is a phosphatidylinositol kinase existing in many kinds of cancer cells and can be activated by a range of cytokine receptors including insulin receptor, growth factor receptor, proto oncogene encoding, and G protein-coupled receptor. PI3K possesses phospholipid kinase activity as well as serine/threonine-protein kinase activity. As the core of the PI3K/AKT signal transduction pathway, AKT can be activated by PI3K, and the prolonged activation is closely related to occurrence and development of tumor. Through phosphorylation, AKT is able to activate a variety of downstream target proteins and subsequently regulates cell proliferation, differentiation, and apoptosis. Glycogen synthase kinase-3*β* (GSK3*β*), one of the major components of the Wnt/*β*-catenin signaling pathway, is the major target protein of AKT. GSK3*β* can be phosphorylated by p-AKT and phosphorylated GSK3*β* can inhibit the degradation of *β*-catenin [27, 28]. Consequently, *β*-catenin accumulates in cytoplasm and is transferred into nucleus to activate downstream genes (Figure 9). In our study, in order to clarify the mechanism of CWP inhibiting gastric cancer, we observed the effect of CWP on gastric cancer based on PI3K/AKT/GSK3*β*/*β*-catenin signaling pathway. Cell experiments indicated that CWP can inhibit proliferation, invasion, and migration of gastric cancer cells when compared with the control group, which is in consistency with clinical observation. We found that CWP can also downregulate the expressions of p-AKT, p-GSK3*β*, and *β*-catenin in a drug dosage-dependent manner. After treatment with IGF-1, agonist of PI3K/AKT, the abilities of invasion and migration are enhanced and the expressions of p-AKT, p-GSK3*β*, and *β*-catenin in cytoplasm and nucleus are increased. On the contrary, PI3K/AKT inhibitor LY294002 can inhibit the invasion of gastric cancer cells and migration, which contributes to downexpression of p-AKT, p-GSK3*β*, and *β*-catenin in the cytoplasm and nucleus. If pretreated with LY294002, neither CWP nor PGE2 could regulate the protein expressions of p-AKT, p-GSK3*β*, and *β*-catenin. Thus, we suggest that CWP inhibits the invasion and metastasis of SGC-7901 cells via Cox-2/PGE2-PI3K/AKT/GSK3*β*/*β*-catenin signaling pathway.

MMPs are the downstream genes of *β*-catenin and they are the key family of proteolytic enzymes involved in the tumor invasion [29]. MMP-2 is one of the important enzymes for degrading type IV collagen in the invasion and metastasis of gastric cancer. MMP-7 is related to tumor invasion and poor prognosis [12, 13]. MMP-14 also has a central role in tumor invasion and not only degrades the ECM but also promotes the secretion of pro-MMP-2. Studies showed that MMP-14 is elevated in gastric cancer patients, and overexpression of MMP-14 is closely associated with gastric cancer invasion [14–16]. CWP can reduce expression levels of MMP-2, MMP-7, and MMP-14, which may be related to mechanism of CWP's antimetastasis effect.

## 5. Conclusion

In conclusion, based on Cox-2/PGE2 relationship, our study has explored possible mechanism of CWP in treating gastric cancer metastasis. Experimental data demonstrates that Cox-2/PGE2 can activate the PI3K/AKT/GSK3*β*/*β*-catenin pathway and CWP can inhibit the invasion and metastasis of gastric cancer by downregulating expression of Cox-2/PGE2. Therefore, we have concluded that CWP may constrain migratory capacity in SGC-7901 cells via Cox-2/PGE2-PI3K/AKT/GSK3*β*/*β*-catenin signaling axis.

## Figures and Tables

**Table 1:** Assay results for Compound Wumei Powder.

Identification	Content of analyte (*μ*g/mL)
Schisandrin	140.65
Chlorogenic acid	123.12
Schizandrin A	8.32
Ferulic acid	1.89

## Abbreviations

IGF-1: Insulin-like growth factor-1
PGE2: Prostaglandin E2
IHC: Immunohistochemical
ELISA: Enzyme-linked immunosorbent assay
PCR: Polymerase Chain Reaction
TCM: Traditional Chinese Medicine
EDTA: Ethylenediaminetetraacetic acid
TBST: Tris-buffered saline with Tween-20
LC-MS/MS: Liquid chromatograph-mass spectrometer/mass spectrometer
DMSO: Dimethyl sulfoxide
PBS: Phosphate-Buffered Saline
HRP: Human Resources Procedure
IOD: Integrated optical density
FN: Fibronectin
PVDF: Polyvinylidene fluoride
SD: Standard deviation
ECM: Extracellular matrix
PI3K: Phosphatidylinositol 3-kinase
GSK3*β*: Glycogen synthase kinase-3*β*
MMP: Matrix metalloproteinase
EP receptor: E-series of Prostaglandin Receptor.

## References

[1] Chen W., Zheng R., Baade P. D. (2016). Cancer statistics in China, 2015. *CA: A Cancer Journal for Clinicians*.

[2] Kang W.-M., Meng Q.-B., Yu J.-C., Ma Z.-Q., Li Z.-T. (2015). Factors associated with early recurrence after curative surgery for gastric cancer. *World Journal of Gastroenterology*.

[3] Song J., Su H., Zhou Y.-Y., Guo L.-L. (2014). Cyclooxygenase-2 expression is associated with poor overall survival of patients with gastric cancer: a meta-analysis. *Digestive Diseases and Sciences*.

[4] Misra S., Sharma K. (2014). COX-2 signaling and cancer: new players in old arena. *Current Drug Targets*.

[5] Lian S., Xia Y., Ung T. T. (2017). Prostaglandin E2 stimulates urokinase-type plasminogen activator receptor via EP2 receptor-dependent signaling pathways in human AGS gastric cancer cells. *Molecular Carcinogenesis*.

[6] Dai J., Qian C., Su M., Chen M., Chen J. (2016). Gastrokine-2 suppresses epithelial mesenchymal transition through PI3K/AKT/GSK3*β* signaling in gastric cancer. *Tumor Biology*.

[7] Hu W., Xiao L., Cao C., Hua S., Wu D. (2016). UBE2T promotes nasopharyngeal carcinoma cell proliferation, invasion, and metastasis by activating the AKT/GSK*β*/*β*-catenin pathway. *Oncotarget *.

[8] Son Y.-O., Wang L., Poyil P. (2012). Cadmium induces carcinogenesis in BEAS-2B cells through ROS-dependent activation of PI3K/AKT/GSK-3*β*/*β*-catenin signaling. *Toxicology and Applied Pharmacology*.

[9] Zhang B., Yin C., Li H. (2013). Nir1 promotes invasion of breast cancer cells by binding to chemokine (C-C motif) ligand 18 through the PI3K/Akt/GSK3*β*/Snail signalling pathway. *European Journal of Cancer*.

[10] Zhao L., Miao H.-C., Li W.-J. (2016). LW-213 induces G2/M cell cycle arrest through AKT/GSK3*β*/*β*-catenin signaling pathway in human breast cancer cells. *Molecular Carcinogenesis*.

[11] Kim N., Kim C. H., Ahn D.-W. (2009). Anti-gastric cancer effects of celecoxib, a selective COX-2 inhibitor, through inhibition of Akt signaling. *Journal of Gastroenterology and Hepatology*.

[12] Mori M., Mimori K., Shiraishi T. (1997). Analysis of MT1-MMP and MMP2 expression in human gastric cancers. *International Journal of Cancer*.

[13] Sakamoto N., Naito Y., Oue N. (2014). MicroRNA-148a is downregulated in gastric cancer, targets MMP7, and indicates tumor invasiveness and poor prognosis. *Cancer Science*.

[14] Zarrabi K., Dufour A., Li J. (2011). Inhibition of Matrix Metalloproteinase 14 (MMP-14)-mediated cancer cell migration. *The Journal of Biological Chemistry*.

[15] Dong Y., Chen G., Gao M., Tian X. (2015). Increased expression of MMP14 correlates with the poor prognosis of Chinese patients with gastric cancer. *Gene*.

[16] Takino T., Watanabe Y., Matsui M. (2006). Membrane-type 1 matrix metalloproteinase modulates focal adhesion stability and cell migration. *Experimental Cell Research*.

[17] Li W., Mao Z., Fan X., Cui L., Wang X. (2014). Cyclooxygenase 2 promoted the tumorigenecity of pancreatic cancer cells. *Tumor Biology*.

[18] Shalaby M. A., Nounou H. A., Alanazi M. S., Alharby O., Azzam N., Saeed H. M. (2014). Associations between single nucleotide polymorphisms of COX-2 and MMP-2 genes and colorectal cancer susceptibility in the saudi population. *Asian Pacific Journal of Cancer Prevention*.

[19] Xin X., Majumder M., Girish G. V., Mohindra V., Maruyama T., Lala P. K. (2012). Targeting COX-2 and EP4 to control tumor growth, angiogenesis, lymphangiogenesis and metastasis to the lungs and lymph nodes in a breast cancer model. *Laboratory Investigation*.

[20] Du M., Shi F., Zhang H. (2015). Prostaglandin E2 promotes human cholangiocarcinoma cell proliferation, migration and invasion through the upregulation of *β*-catenin expression via EP3-4 receptor. *Oncology Reports*.

[21] Liu J., Zhang Y., Xu R. (2013). PI3K/Akt-dependent phosphorylation of GSK3*β* and activation of RhoA regulate Wnt5a-induced gastric cancer cell migration. *Cellular Signalling*.

[22] Chan A. T., Giovannucci E. L., Meyerhardt J. A., Schernhammer E. S., Wu K., Fuchs C. S. (2008). Aspirin dose and duration of use and risk of colorectal cancer in men. *Gastroenterology*.

[23] Farooqui M., Li Y., Rogers T. (2007). COX-2 inhibitor celecoxib prevents chronic morphine-induced promotion of angiogenesis, tumour growth, metastasis and mortality, without compromising analgesia. *British Journal of Cancer*.

[24] Ni C.-X., Qi Y., Zhang J. (2016). WM130 preferentially inhibits hepatic cancer stem-like cells by suppressing AKT/GSK3*β*/*β*-catenin signaling pathway. *Oncotarget *.

[25] Sun Y., Gao C., Luo M. (2013). Aspidin PB, a phloroglucinol derivative, induces apoptosis in human hepatocarcinoma HepG2 cells by modulating PI3K/Akt/GSK3*β* pathway. *Chemico-Biological Interactions*.

[26] Wang G., Feng C.-C., Chu S.-J. (2015). Toosendanin inhibits growth and induces apoptosis in colorectal cancer cells through suppression of AKT/GSK-3*β*/*β*-catenin pathway. *International Journal of Oncology*.

[27] Bishnupuri K. S., Sainathan S. K., Bishnupuri K. (2014). Reg4-induced mitogenesis involves Akt-GSK3*β*-*β*-Catenin-TCF-4 signaling in human colorectal cancer. *Molecular Carcinogenesis*.

[28] Wu J., Zhang H., Xu C. (2016). TIPE2 functions as a metastasis suppressor via negatively regulating catenin through activating GSK3 in gastric cancer. *International Journal of Oncology*.

[29] Velinov N., Poptodorov G., Gabrovski N., Gabrovski S. (2010). The role of matrixmetalloproteinases in the tumor growth and metastasis. *Khirurgiia*.

